# Efficacy of immune checkpoint inhibitors in the treatment of non-small cell lung cancer patients with different genes mutation: A meta-analysis

**DOI:** 10.1097/MD.0000000000019713

**Published:** 2021-03-12

**Authors:** Rui Zhang, Jing Zhu, Ying Liu, Ying Xin, Ying Wang, Kai Niu, Huafang Wei

**Affiliations:** Department of thoracic Oncology, JiLin Province Cancer Hospital, Changchun, JiLin, PR China.

**Keywords:** epidermal growth factor receptor, KRAS, meta-analysis, non-small cell lung cancer, PD-L1, sensitive genes mutation

## Abstract

**Background:**

Latest clinical trials have proved the better overall survival (OS) for the use of immune checkpoint inhibitors verse chemotherapy in non-small cell lung cancer (NSCLC) patients. However, we still have no clear ideas of the factors which could affect the efficacy of immune checkpoint inhibitors. Cancer, essentially, is a disease related to genes mutation. Therefore, we conducted a systematic review and meta-analysis to compare efficacy of immune checkpoint inhibitors for NSCLC patients with different genes mutation.

**Methods:**

PubMed, EMBASE, Web of Science, and the Cochrane Library databases were searched for all clinical trials in NSCLC until December 16, 2019. The hazard ratio (HR) and 95% confidence intervals (CIs) of OS or progression-free survival (PFS) were used.

**Results:**

A total of 4453 patients from 7 randomized controlled trials (RCTs) were included. Immune checkpoint inhibitors significantly prolonged the OS (HR, 0.67; 95% CI, 0.60–0.67) in NSCLC patients having epidermal growth factor receptor (EGFR) wild-type versus chemotherapy. Meanwhile, they prolonged the OS (HR, 0.61; 95% CI, 0.39–0.94) in NSCLC patients with Kirsten rat sarcoma viral oncogene homolog (KRAS) mutation. No matter PD-L1 tumor proportion scores were >1% or <1%, immune checkpoint inhibitors were more effective than chemotherapy (HR, 0.64; 95% CI, 0.55–0.75).

**Conclusion:**

Immune checkpoint inhibitors are more efficacious than chemotherapy in NSCLC patients with EGFR wild-type, KRAS mutation, and any PD-L1 tumor proportion scores.

## 1 Introduction

Lung cancer has the highest mortality rate among all the tumors and essentially it is a disease related to abnormal genes mutation. For non-small cell lung cancer (NSCLC) patients having certain sensitive genes mutation (such as epidermal growth factor receptor [EGFR], anaplastic lymphoma kinase [ALK], Kirsten rat sarcoma viral oncogene homolog (KRAS), and so on), molecularly targeted therapies have transformed treatment and improved 5 years survival rate substantially.^[1,2]^ Recently, with the increasing use of immune checkpoint inhibitors in large randomized controlled trials, the immune therapy has gradually turned into the mainstream cancer therapy.^[3,4]^ The monoclonal antibodies against cytotoxic T-lymphocyte-associated protein 4 (CTLA-4) and PD-1 have been the best studied immune therapies so far.^[5]^ As to the squamous NSCLC Nivolumab and Pembrolizumab which are the antibodies against PD-1 have become the first line therapy superior to chemotherapy.^[3,6]^

However, we still have no clear idea of the factors which could affect the efficacy of immune checkpoint inhibitors. Also, how to apply targeted drugs, chemotherapy, and immune therapy in different patients has been a clinical problem. At present, a few subgroups of clinical trials have reported the comparison between immune checkpoint inhibitors and chemotherapy in patients with different molecular characteristics, such as PD-L1 protein expression,^[7]^ EGFR gene expression,^[8,9]^ tumor mutational burden (TMB),^[10]^ and so on. In order to explore the most suitable patients for immune therapy, we conducted a systematic review to explore the efficacy of immune checkpoint inhibitors for NSCLC patients with different genes mutation. We hope to provide more definite evidence for clinicians to choose targeted patients for immune checkpoint inhibitors.

## 2 Materials and methods

### 2.1 Literature search

This systematic review was reported in accordance with the preferred reporting items for systematic reviews and meta-analyses (PRISMA) statement.^[11]^ PubMed, Web of Science, EMBASE, and the Cochrane Central Register of Controlled Trials (Central) databases were searched for potentially relevant studies until December 16, 2019.

We searched studies from these databases in all fields with “Nivolumab” OR “Opdivo” OR “ONO-4538” OR “MDX-1106” OR “BMS-936558” OR “Ipilimumab” OR “Yervoy” OR “MDX-010” OR “MDX-CTLA-4” OR “Pembrolizumab” OR “Keytruda” OR “Lambrolizumab” OR “MK-3475” OR “Atezolizumab” OR “MPDL3280A” OR “Tecentriq” OR “RG-7446” OR “Durvalumab” OR “MEDI-4736” OR “Imfinzi” OR “Avelumab” OR “MSB0010718C” OR “PD-1” OR “PD-L1” OR“PD-1/PD-L1” OR “programmed cell death 1” OR “programmed cell death ligand 1” AND “Carcinoma, Non-Small Cell Lung” OR “Lung Carcinoma, Non-Small-Cell” OR “Non-Small Cell Lung Cancer” as the keywords. Articles that were not published in English were excluded.

### 2.2 Selection criteria

The inclusion criteria were listed as follows: the object of the trial should be NSCLC patients. The intervention ought to be include PD-1, PD-L1, or CTLA-4 inhibitors. The control group ought to be treated with chemotherapy. The outcome of overall survival (OS) or progression-free survival (PFS) for NSCLC patients having sensitive genes mutation (such as EGFR, ALK, KRAS, PD-L1, and so on) should be reported. The trials should be Phase III or Phase II/III randomized controlled trials (RCTs). The following excluded criteria were used: no chemotherapy control arm; studies not in English.

### 2.3 Data extraction

The data were extracted by 2 authors independently. The following information were extracted from the trials: first author, year of publication, histology of lung cancer, therapeutic line, trial phase, number of patients, experimental arms, control arms, hazard ratio (HR) of PFS or OS. The third author assessed the data and resolved the disagreement.

### 2.4 Assessment of study quality and publication bias

The risk of bias was assessed according to the Cochrane Handbook for Systematic Reviews of Interventions,^[12]^ which involves assessing bias relating to random sequence generation, allocation concealment, blinding, data integrity, selective reporting of positive and/or negative findings, and other sources of bias. Among the “other sources of bias” included: were there clear inclusion/exclusion criteria; were the baseline data comparable; and was there any conflict of interest. All the included clinical studies have been registered. The risk of bias was assessed and validated independently by 3 authors; the results were cross-referenced, and any disagreements were resolved by discussion with a third author.

### 2.5 Statistical analysis

Review Manager 5.3 (Cochrane Collaboration; London, United Kingdom) was used to conduct our systematical review, making forest plots. Confidence interval (CI) and HR were used as effect sizes. In this analysis, if *P* for heterogeneity was <.10 or *I*^2^ > 50%, the null hypothesis that the studies were homogenous would be rejected. When there was significant heterogeneity among the results of included study, the random effects model was used to calculate summary estimate.^[13]^ Otherwise, the summary estimate would be based on the fixed effects model. It was reported using the opposite variance method, supposing that the studies included had the same effect size. Publication bias was shown by funnel plot.

## 3 Results

### 3.1 Search results and patients characteristics

**Figure 1:**
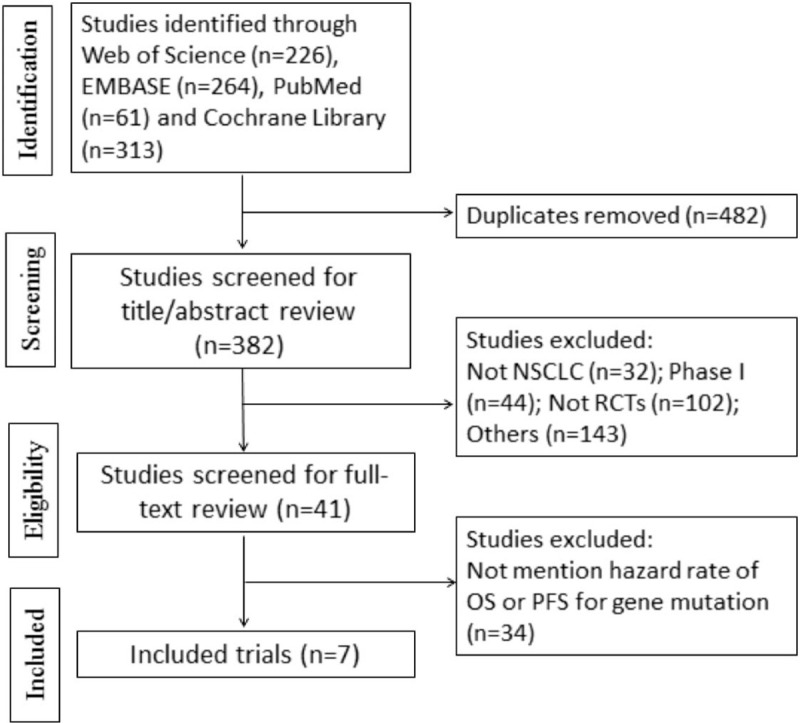
Flow diagram: selection process for the studies. NSCLC = non-small cell lung cancer, OS = overall survival, PFS = progression-free survival, RCT = randomized controlled trial.

Of the 864 identified trials, 7 were included. The screening flow chart was shown in Fig. 1. A total of 4453 patients were recorded in these studies.^[14–20]^ Three^[14,16,18]^ trials evaluated the effectiveness of immune checkpoint inhibitors for NSCLC patients having EGFR mutation or wild-type, including PD-1 (n = 915), PD-L1 (n = 713). Two trials^[14,18]^ evaluated the effect of PD-1 and PD-L1 inhibitors for NSCLC patients having KRAS mutation or wild-type, including PD-1 (n = 185), PD-L1 (n = 262). The characteristics of the RCTs were shown in Table 1. (Nivolumab and Pembrolizumab are all human IgG4 PD-1 immune checkpoint inhibitor antibodies. Due to the comparison of famous RCTs between Checkmate 057 and KEYNOTE 010, it has been proved that the patents treated by them would have similar OS.^[21]^ So we choose to regard them as a group for discussion.)

**Table 1:** The characteristics of the included studies.

Author, year	Line	Phase	Histology	Stage	Experimental arms(s)	Immune target	Control arms(s)	Number	Follow-up, mo	CTCAE version
H. Borghaei, 2015	Non first-line	III	Non-squamous NSCLC	IIIB or IV	Nivolumab	PD-1	Docetaxel	582	18	4.0
J. Brahmer, 2015	Non first-line	III	Squamous NSCLC	IIIB or IV	Nivolumab	PD-1	Docetaxel	272	20.5	4.0
Roy S. Herbst,2016	Non first-line	II/III	NSCLC	IV	Pembrolizumab	PD-1	Docetaxel	1033	17.3	4.0
D.P. Carbone, 2017	First-line	III	NSCLC	IV	Nivolumab	PD-1	Chemotherapy	541	17.4	4.0
Achim Rittmeyer, 2017	Non first-line	III	NSCLC	IIIB or IV	Atezolizumab	PD-L1	docetaxel	850	21	4.0
L. Gandhi, 2018	First-line	II	Non-squamous NSCLC	IV	Pembrolizumab + chemotherapy	PD-1	Chemotherapy	616	20.4	4.0
L. Paz-Ares, 2018	First-line	III	Squamous NSCLC	IV	Pembrolizumab + chemotherapy	PD-1	Chemotherapy	559	19.1	4.0

NSCLC = non-small cell lung cancer.

### 3.2 Evaluation of study quality and publication bias

**Figure 2:**
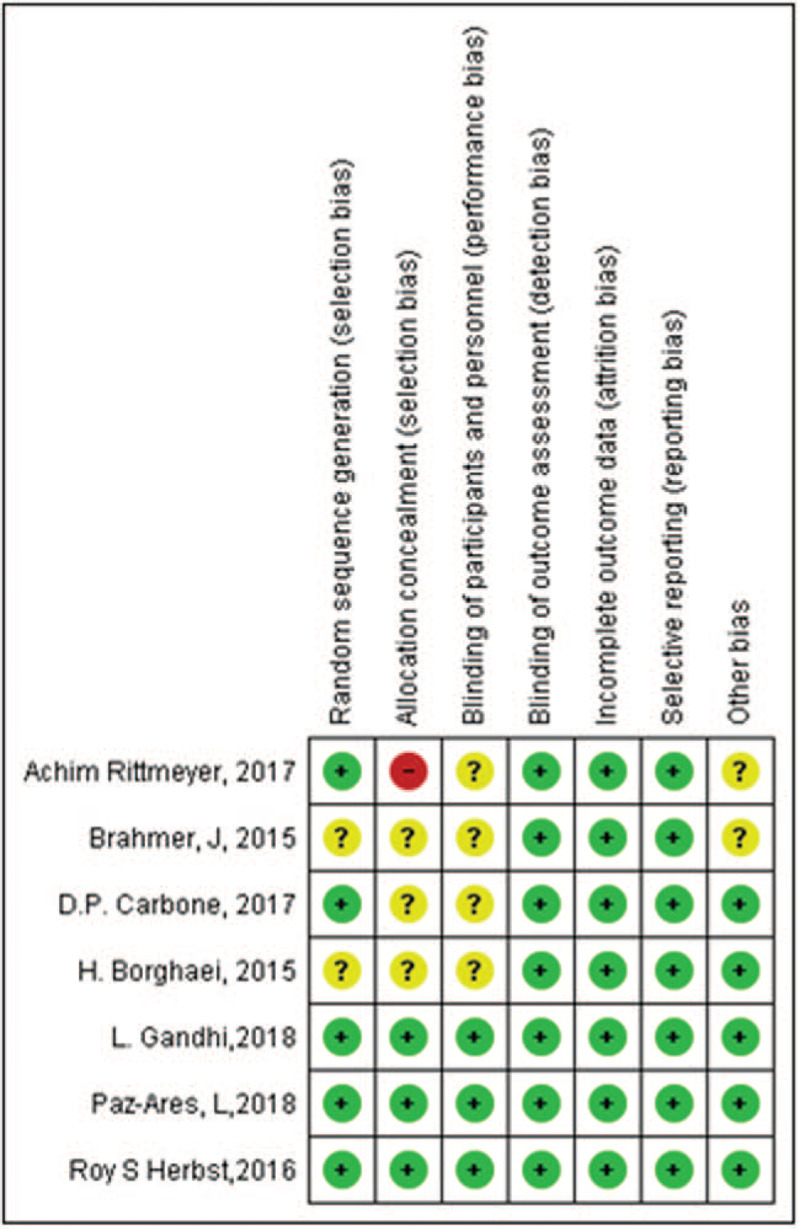
The assessment of risk of bias.

**Figure 3:**
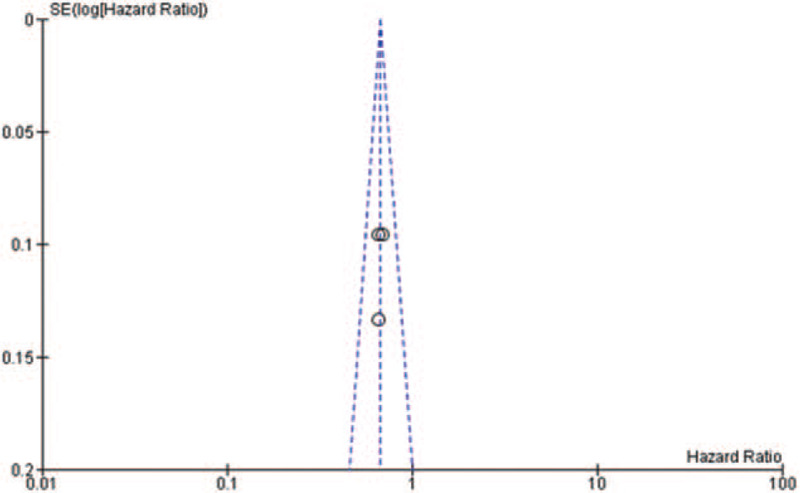
The funnel plot of studies evaluating EGFR expression. EGFR = epidermal growth factor receptor.

**Figure 4:**
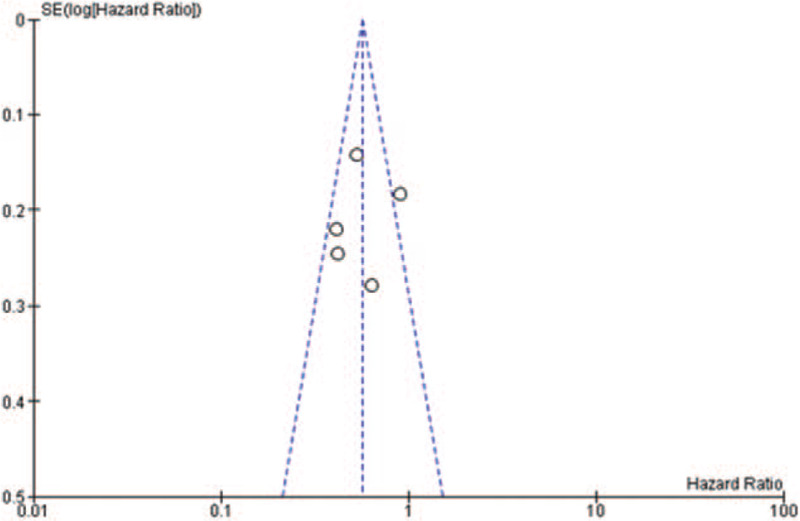
The funnel plot of studies evaluating PD-L1 tumor proportion scores.

Some of the included studies had a high^[18]^ or unclear risk^[14–15,17]^ of selection bias, due to the difficulty in the process of allocation concealment. All of them had a comparatively complete report of the outcome data. The assessment of risk of bias was shown in Fig. 2. There was no publication bias observed in those studies evaluating gene EGFR and the funnel plot was shown in Fig. 3. There might be little publication bias observed in those studies evaluating gene PD-L1 and the funnel plot was shown in Fig. 4.

### 3.3 EGFR mutation or wild-type

**Figure 5:**
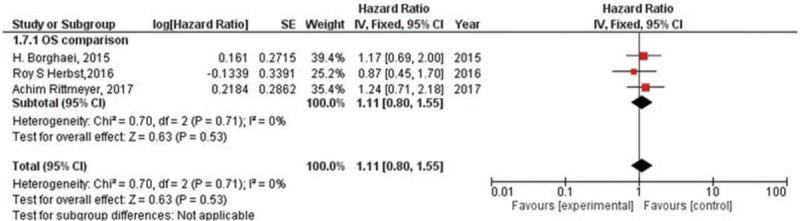
Forest plot for OS of NSCLC patients with EGFR mutation. EGFR = epidermal growth factor receptor, OS = overall survival, NSCLC = non-small cell lung cancer.

**Figure 6:**
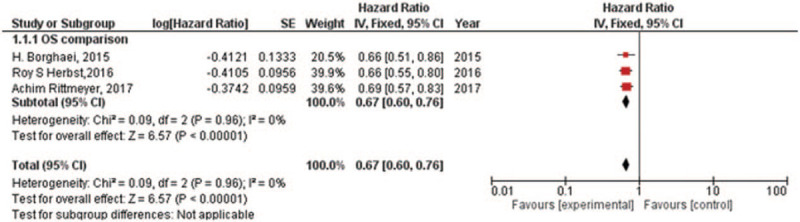
Forest plot for OS NSCLC patients with EGFR wild-type. EGFR = epidermal growth factor receptor, OS = overall survival, NSCLC = non-small cell lung cancer.

There were 3 RCTs which compared the OS^[14,16,18]^ for NSCLC with EGFR mutation or wild-type. For EGFR mutation, there was low heterogeneity in OS (*I*^2^ = 0%) analysis. Hence, fixed-effect model was used in this analysis. The meta-analysis showed (Fig. 5) that immune checkpoint inhibitors versus chemotherapy had similar effects in treating NSCLC patients having EGFR mutation for the OS (HR, 1.11; 95% CI, 0.80–1.55). For EGFR wild-type, there was still low heterogeneity in total (*I*^2^ = 0%) analysis. Hence, fixed-effect model was used in this analysis. The meta-analysis showed (Fig. 6) that immune checkpoint inhibitors versus chemotherapy had prolonged the NSCLC patients having EGFR wild-type for the OS (HR, 0.67; 95% CI, 0.60–0.76).

### 3.4 KRAS mutation or wild-type

**Figure 7:**
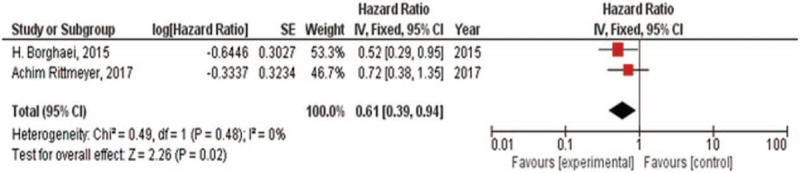
Forest plot for OS and PFS of NSCLC patients with KRAS mutation. NSCLC = non-small cell lung cancer, OS = overall survival, PFS = progression-free survival.

**Figure 8:**
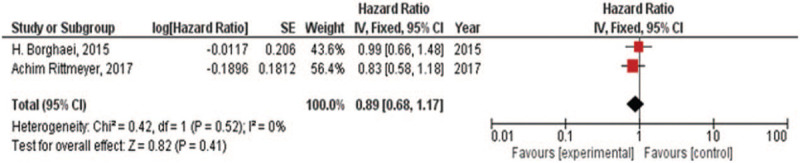
Forest plot for OS and PFS of NSCLC patients with KRS wild-type. NSCLC = non-small cell lung cancer, OS = overall survival, PFS = progression-free survival.

There were 2 RCTs which compared the OS^[14,18]^ for NSCLC with KRAS mutation or wild-type. For KRAS mutation, there was low heterogeneity in OS (*I*^2^ = 0%) analysis. Hence, fixed-effect model was used in this analysis. The meta-analysis showed (Fig. 7) that Immune checkpoint inhibitors versus chemotherapy had prolonged the NSCLC patients with KRAS mutation for the OS (HR, 0.61; 95% CI, 0.39–0.94). For KRAS wild-type, there was either low heterogeneity in OS (*I*^2^ = 0%) analysis. Hence, fixed-effect model was also used in this analysis. The meta-analysis showed (Fig. 8) that immune checkpoint inhibitors versus chemotherapy had similar effects in treating NSCLC patients having KRAS wild-type for the OS (HR, 0.89; 95% CI, 0.68–1.17).

### 3.5 Different PD-L1 mutation tumor proportion scores

There were 6 RCTs which compared the OS^[15–20]^ and PFS^[15–20]^ for NSCLC with different PD-L1 tumor proportion scores. These studies classified PD-L1 tumor proportion scores into different groups, including less or >1%, less or >5%, less or >10%, >50%, from 1% to 49% and not quantifiable. We used random-effects model and fixed-effects model to merge similar grouping together and the HR for the OS was shown in Table 2, the HR for the PFS shown in Table 3.

**Table 2:** Meta-analysis of pooled hazard ratios and 95% CI of overall survival for patients with different PD-L1 tumor proportion scores.

		Random-effects model		Fixed-effects model		Heterogeneity	
PD-L1 tumor proportion scores	N	HR (95% CI)	*P*	HR (95% CI)	*P*	*I*^2^	*P*
≥1%	4	0.63 (0.52, 0.77)	<.00001	0.64 (0.55, 0.75)	<.00001	34%	.21
<1%	4	0.68 (0.56, 0.81)	<.0001	0.68 (0.56, 0.81)	<.0001	0%	.64
≥5%	2	0.63 (0.48–0.82)	.0005	0.63 (0.48–0.82)	.0005	0%	.45
<5%	1	0.70 (0.47–1.02)	.06	0.70 (0.47–1.02)	.06	Not applicable	
≥10%	1	0.50 (0.28–0.89)	.02	0.50 (0.28–0.89)	.02	Not applicable	
<10%	1	0.70 (0.48–1.01)	.06	0.70 (0.48–1.01)	.06	Not applicable	
≥50%	5	0.56 (0.42, 0.75)	.0001	0.57 (0.48, 0.68)	<.00001	61%	.04
1–49%	3	0.68 (0.56, 0.83)	.0002	0.69 (0.57, 0.83)	.0001	5%	.35
Not quantifiable	1	0.39 (0.19–0.82)	.01	0.39 (0.19–0.82)	.01	Not applicable	

CIs = confidence intervals, HR = hazard ratio.

**Table 3:** Meta-analysis of pooled hazard ratios and 95% CI of progression-free survival for patients with different PD-L1 tumor proportion scores.

		Random-effects model		Fixed-effects model		Heterogeneity	
PD-L1 tumor proportion scores	N	HR (95% CI)	*P*	HR (95% CI)	*P*	*I*^2^	*P*
≥1%	3	0.50 (0.41, 0.62)	.25	0.50 (0.42, 0.59)	<.00001	28%	.25
<1%	3	0.70 (0.57, 0.87)	.001	0.70 (0.57, 0.87)	.001	0%	.90
≥5%	1	0.54 (0.32–0.90)	.02	0.54 (0.32–0.90)	.02	Not applicable	
<5%	1	0.75 (0.52–1.08)	.12	0.75 (0.52–1.08)	.12	Not applicable	
≥10%	1	0.58 (0.33–1.02)	.06	0.58 (0.33–1.02)	.06	Not applicable	
<10%	1	0.70 (0.49–0.99)	.04	0.70 (0.49–0.99)	.04	Not applicable	
≥50%	4	0.55 (0.34, 0.87)	.01	0.82 (0.64, 1.04)	<.00001	87%	<.0001
1–49%	3	0.73 (0.61, 0.89)	.14	0.76 (0.69, 0.84)	.02	86%	.001
Not quantifiable	1	0.45 (0.23–0.89)	.02	0.45 (0.23–0.89)	.02	Not applicable	

CIs = confidence intervals, HR = hazard ratio.

## 4 Discussion

At present, immune therapy has gradually come into the mainstream cancer therapy. The monoclonal antibodies against PD-1 have been the best studied immune therapies so far.^[5]^ As we all know, PD-L1 is the ligand of PD-1, which is often expressed in cancer cells. The cancer cells may escape the anti-tumor effect of T cells by over-expression of the PD-L1 in the tumor microenvironment. Hence, using immune checkpoint inhibitors that block the increasing PD-L1 molecules might be able to prevent tumor cells from evading human body.^[22]^ In summary, immune checkpoint inhibitors work by activating human immune system to kill tumors. Recently, an increasing number of prospective trials and meta-analysis^[23,24]^ have proved the efficacy of immune checkpoint inhibitors in NSCLC patients compared with chemotherapy. It is worth noting that once immune checkpoint inhibitors work they are more likely to keep its function of anti-cancer for a long time,^[25]^ and some people could even be completely cured. However, the suitable patients who could achieve significant improvement from immune therapy are still being explored.

Cancer, in essence, is a disease related to genes mutation. Gene mutations in the EGFR are detected in 10% to 15% of NSCLCs from Caucasian patients and about 30% to 40% from Asian patients,^[26]^ ALK overexpressed in 5% of NSCLCs.^[27]^ Due to the long 5-year survival rate of applying targeted drugs, immune therapy has been mostly applied in NSCLC patients without sensitive genes mutation, mainly the squamous cell lung carcinoma.^[28,29]^ But targeted drugs will be resistant as the time goes. As a result, some new questions are raised: will immune checkpoint inhibitors be new choices for these targeted drugs resistant NSCLCs or will these genes expression affect the efficacy of immune therapy?

The meta-analyses^[30,31]^ of early trials have reviewed the efficacy of immune checkpoint inhibitors in NSCLC patients having EGFR mutation. Complementarily, our group reviewed the subgroups including different genes expression of RCTs, such as EGFR, KRAS, and PD-L1. It proved that immune checkpoint inhibitors might have no difference (the OS of HR, 1.11; 95% CI, 0.80–1.55) compared with chemotherapy for NSCLC patients having EGFR mutation. Meanwhile, immune checkpoint inhibitors could be a recommendation for NSCLC patients having EGFR wild-type (the OS of HR, 0.67; 95% CI, 0.60–0.76). We also found immune checkpoint inhibitors appear to be more effective in NSCLC patients having KRAS mutation (the OS of HR, 0.61; 95% CI, 0.39–0.94) compared with chemotherapy and no difference in NSCLC patients having KRAS wild-type (the OS of HR, 0.89; 95% CI, 0.68–1.17). Due to mutations in EGFR and KRAS appearing to be mutually exclusive in lung cancer patients,^[32]^ the results are especially worth noticing. It is a pity that some trials^[14,15,17]^ recorded the status of ALK in the baseline but no comparison of their OS data between immune checkpoint inhibitors and chemotherapy. Furthermore, we reviewed the OS and PFS for patients with different PD-L1 tumor proportion scores. Except from the analysis only including one trial, no matter PD-L1 tumor proportion scores >1% or <1% or not quantifiable, they all showed that immune therapy was more effective than chemotherapy.

According to the meta-analyses, we have found that the patients with EGFR wild-type or KRAS mutation could achieve better OS from the immune checkpoint inhibitors. And the efficacy of immune therapy has no relation to PD-L1 expression when it is compared with chemotherapy, but it seems that the efficacy have improved with PD-L1 expression increasing. Some literatures^[8,33]^ reported that PD-L1 expression was reduced in NSCLC cell lines by activating EGFR, meanwhile some researchers^[34]^ found that EGFR—tyrosine kinase inhibitors (TKIs) could indirectly enhance anti-tumor immunity through the down-regulation of PD-L1, which might explain the reverse effect of EGFR and PD-L1. Therefore, immune therapy or combination of immune therapy and targeted drugs might not be recommended when patients have sensitive genes mutation. Yet someone reported that PD-L1 antibodies could be an optional therapy for EGFR mutant NSCLC without targetable resistant mutations^[35]^ due to the YAP (yes-associated protein) mediating, which needs further research.

In our opinions, more details of immune checkpoint inhibitors treating NSCLC patients with different genes mutation, not only EGFR or KRAS, but also ALK, BRAF and the like, in RCTs need to be complementarily reported. And the mechanism of interactions those genes mutation needs to be further explored for better clinical application.

## 5 Conclusion

In conclusion, immune checkpoint inhibitors could be a recommendation for NSCLC patients having EGFR wild-type, KRAS mutation, and PD-L1 tumor proportion scores >1%. More details of immune checkpoint inhibitors treating NSCLC patients with sensitive gene mutation (not only EGFR and KRAS, but also ALK, BRAF, and the like) in some open-label multicenter randomized controlled trials need to be reported complementarily. How to use immune checkpoint inhibitors efficiently in NSCLC patients with sensitive genes mutation is worth further exploring.

## Author contributions

All the authors took part in the search of literature. The data of the selected trials were extracted by 2 authors (JZ and RZ) independently. Three authors (YX, YW, and KN) used the Cochrane Collaboration's risk of bias tool to assess the methodological quality of the included studies. JZ, RZ, and YL wrote the paper.
